# Blood Type A1 Mismatch Does Not Affect Heart Transplant Outcomes at One Year

**DOI:** 10.3390/jcm12041337

**Published:** 2023-02-08

**Authors:** Louie Cao, Seongkyu Kim, Ellen Klapper, Jon A. Kobashigawa, Michelle M. Kittleson

**Affiliations:** 1 Department of Internal Medicine, Cedars-Sinai Medical Center, Los Angeles, CA 90048, USA; 2 Department of Cardiology, Smidt Heart Institute, Cedars-Sinai Medical Center, Los Angeles, CA 90048, USA; 3 The Division of Transfusion Medicine, Department of Pathology and Laboratory Medicine, Cedars-Sinai Health System, Los Angeles, CA 90048, USA

**Keywords:** heart transplant, ABO subtype, rejection

## Abstract

There are subtypes within blood type A, termed non-A1, that have reduced expression of A antigen on cell surfaces. This can result in the development of anti-A1 antibodies. There is limited information regarding the impact of this in heart transplant (HTx) recipients. We conducted a single-center cohort study of 142 Type A HTx recipients in which we compared outcomes of a match group (an A1/O heart into an A1 recipient or a non-A1/O heart into a non-A1 recipient) with a mismatch group (an A1 heart into a non-A1 recipient or a non-A1 heart into an A1 recipient). At one year post-transplant, there were no differences between the groups in survival, freedom from non-fatal major adverse cardiovascular events, freedom from any treated rejection, or freedom from cardiac allograft vasculopathy. There was an increased hospital length of stay in the mismatch group (13.5 vs. 17.1 days, *p* = 0.04). Our study showed that A1 mismatch was not associated with worse outcomes at one year post-HTx.

## 1. Introduction

ABO blood type compatibility is a prerequisite for heart transplantation (HTx) due to the risk of hyperacute rejection mediated by ABO antibodies [1]. These antibodies bind specific carbohydrate moieties defining the A and B antigens. However, conventional ABO grouping does not capture inter-individual variation in antigen expression.

Within blood type A there are subtypes, termed non-A1, which have reduced antigen expression and density because of variation in transferase specificity and efficiency. For example, the A2 subgroup, which makes up 20% of group A individuals, expresses fewer A antigen epitopes per red blood cell and is considered less immunogenic [2]. When considering transplantation of an A1 organ to a non-A1 recipient, there is a theoretical risk for adverse outcomes given the potential for interaction of recipient anti-A1 antibodies, if present, with the A1 donor organ. In 2013, an update to the Organ Procurement and Transplantation Network policy stipulated that two ABO results from donors and recipients must be documented, and that confirmation of the A or AB subtype must occur prior to proceeding with transplantation [3].

Research into the safety of organ transplantation across the A1 barrier has been very limited. There are a few case reports of non-A1 recipients with anti-A1 titers of ≤ 1:8 receiving A1 kidneys that did not result in allograft rejection [4,5,6]. A review of the heart transplantation (HTx) literature revealed only one small single-center study with eight non-A1 recipients, none of whom developed anti-A1 antibodies even though five donor hearts were A1. There was no difference in freedom from rejection, graft dysfunction, cardiac allograft vasculopathy (CAV), or re-transplantation at 6 months compared to A1 recipients [7]. We sought to gather further data on the effect of A1-mismatched HTx at our high-volume transplant center. We hypothesized that these would have worse outcomes when compared to A1-matched HTx due to anti-A1 antibodies driving immunologically-mediated phenomena, such as rejection and CAV. However, we found that A1 mismatch did not result in adverse outcomes at one year post-HTx.

## 2. Materials and Methods

This was a single-center cohort study conducted at Cedars-Sinai Medical Center in Los Angeles, California. Adults aged 18 years or older with blood type A who received a HTx between 2013 and 2020 were eligible for inclusion in the study. Subjects were excluded if they had a previous HTx or if they were younger than 18 years old. Subjects’ blood samples were collected and tested for the A1 subtype if there was a discrepancy in blood type testing. Anti-A1 antibody titer testing was performed once, at the time of enrollment, on all subjects who were identified as non-A1. Donor blood types (A versus O) and A subtypes (A1 versus non-A1) were collected from the United Network of Organ Sharing (UNOS).

Desensitization therapy was performed if calculated panel reactive antibodies (cPRA) were greater than 50–70%. All subjects received a standard post-HTx immunosuppression regimen of prednisone, mycophenolate mofetil, and tacrolimus. Blood subtype and the presence/absence of anti-A1 antibodies did not influence desensitization or immunosuppression strategy. Surveillance for rejection via endomyocardial biopsy was performed at protocolized intervals for all subjects regardless of A1 matching. Acute cellular rejection was defined according to the International Society for Heart and Lung Transplantation (ISHLT) 2004 grading system [8]. Antibody-mediated rejection was defined according to the ISHLT 2013 grading system [9]. Cardiac allograft vasculopathy was defined as any angiographic stenosis greater than 30% on a routine surveillance angiogram.

All other demographic and clinical information was collected from chart review utilizing the Cedars-Sinai Electronic Medical Record (EPIC™). To account for the 2018 change in status listings, we defined “urgent status at transplant” as status 1A in the previous scheme and statuses 1, 2, and 3 in the current scheme. Predicted heart mass (PHM) was calculated using a UNOS calculator [10].

Our outcomes of interest were survival, freedom from CAV, freedom from non-fatal major adverse cardiac events (NF-MACE: myocardial infarction, heart failure (HF), percutaneous coronary intervention (PCI), defibrillator/pacemaker implant (ICD/PM), or stroke), and freedom from any treated rejection (ATR), acute cellular rejection (ACR), and antibody-mediated rejection (AMR) at one year post-HTx. We assessed subclinical markers, including ejection fraction (EF) and donor-specific antibodies (DSA), at one year. We also collected immediate post-HTx data including primary graft dysfunction (PGD), vasoplegia, and length of stay (LOS) in the intensive care unit (ICU) and hospital.

Results were compared between a “match” group (an A1/O donor into an A1 recipient or a non-A1/O donor into a non-A1 recipient) and a “mismatch” group (an A1 donor into a non-A1 recipient or a non-A1 donor into an A1 recipient). Continuous variables were reported as mean ± standard deviation and compared using the independent samples t-test. Categorical variables were reported as percentages and compared using Fischer’s exact test. Survival was calculated using the Kaplan–Meier method. All comparisons were two-tailed, and *p*-values < 0.05 were considered significant. Statistical analysis was performed using the data analysis program, SPSS version 24.0 (IBM Corp., Armonk, NY, USA).

The study protocol was approved by the Cedars-Sinai institutional review board (ethical approval code Pro00057683).

## 3. Results

**Figure 1:**
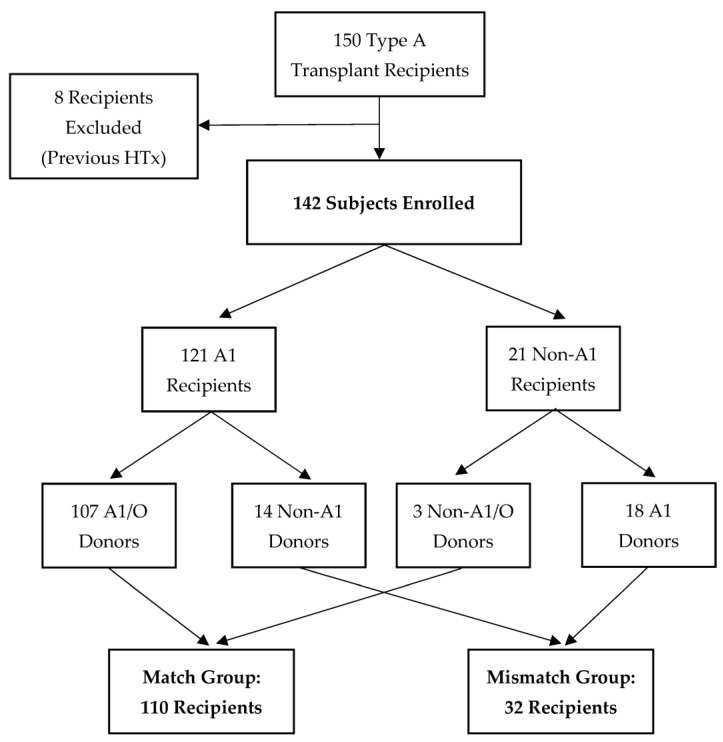
Allocation of Subjects into Match and Mismatch Groups.

We identified 150 patients with blood type A who were transplanted between 2013 and 2020. Of 142 enrolled subjects, 8 were excluded because they had a previous HTx. Subjects were enrolled between 10 days and 6.5 years after HTx. with an average of 2.4 years between HTx and enrollment. Fifty-five (39%) subjects were enrolled within one year post-transplant. Allocation into study groups is depicted in Figure 1; 121 (85%) subjects were identified as A1, and 21 (15%) were identified as non-A1. Of 142 subjects, 110 (77%) were included in the match group as follows: 107 A1/O hearts into A1 recipients and 3 non-A1/O hearts into non-A1 recipients. Of 142 subjects, 32 (23%) were included in the mismatch group: 18 A1 hearts into non-A1 recipients and 14 non-A1 hearts into A1 recipients. None of the non-A1 recipients were found to have anti-A1 antibodies when tested at the time of study enrollment.

### 3.1. Pre-Transplant Clinical Characteristics

Pre-transplant clinical characteristics are shown in Table 1. Between the match and mismatch groups, there was a difference in racial composition, which was driven by a large proportion of patients identifying as “other” (2.7% vs. 18.8%, respectively, *p* = 0.02). All other baseline demographics, including recipient age, donor age, sex, body mass index, and predicted heart mass, were similar between groups. There were no differences in comorbidities such as diabetes, hypertension, or prior cardiac surgery. Both groups had similar types of underlying cardiomyopathy. The two groups had similar risks for pre-transplant sensitization, as indicated by rates of previous pregnancy, previous blood transfusions, PRA with mean fluorescence intensity (MFI) > 5000, pre-transplant desensitization, and induction with anti-thymocyte globulin (ATG). Subjects in the match and mismatch groups had similar waitlist trajectories with no significant difference in time spent on the waitlist. Each group had similar proportions of subjects who were seriously ill, as represented by an urgent transplant status, and had similar kidney function, as measured via creatinine immediately prior to transplant. Ischemic time in the donor heart was similar between groups.

### 3.2. Post-Transplant Outcomes

**Figure 2:**
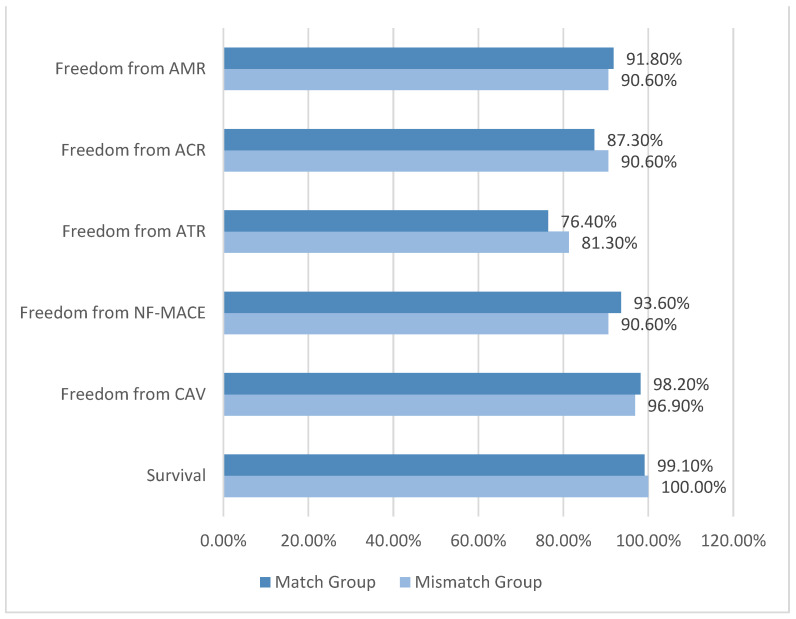
Post-Transplant Outcomes at One Year Post-HTx.

Post-transplant outcomes are shown in Table 2 and Figure 2. There was no difference in outcomes at one year post-transplant, including survival (99.1% in the match group vs. 100% in the mismatch group, *p* = 0.58), freedom from CAV (98.2% vs. 96.9%, *p* = 0.67), and freedom from NF-MACE (93.6% vs. 90.6%, *p* = 0.52). There were seven NF-MACE events in the match group: one HF, one PCI, one ICD/PM, and four strokes. There were three NF-MACE events in the mismatch group: two ICD/PMs and one stroke. The mismatch group did not have an increased incidence of immune-mediated adverse events, with similar rates of freedom from ATR (76.4% vs. 81.3%, *p* = 0.61), freedom from ACR (87.3% vs. 90.6%, *p* = 0.62), and freedom from AMR (91.8% vs. 90.6%, *p* = 0.80) between groups. At a subclinical level, there was no difference in ejection fraction (62.6% vs. 60.9%, *p* = 0.11) or the incidence of donor-specific antibodies (17.3% vs. 9.4%, *p* = 0.41) at one year.

The mismatch group did have a higher post-transplant LOS in the hospital overall (13.5 vs. 17.1 days, *p* = 0.04), but not in the ICU (6.5 vs. 8.0 days, *p* = 0.08). This may have been driven by numerically higher rates of PGD (12.7% vs. 18.8%, *p* = 0.40) and vasoplegia (11.8% vs. 15.6%, *p* = 0.56), although these differences were not statistically significant.

## 4. Discussion

In this study, we divided HTx recipients with blood type A into match and mismatch groups based on the donor and recipient subtypes. These two groups were very similar in terms of demographics, comorbidities, severity of illness while on the transplant waitlist, and risk for sensitization. We did not observe any significant difference in one-year outcomes between these two groups.

The mismatch group was further broken down into two subgroups. For A1 recipients who received non-A1 hearts, we expected comparable outcomes with the match group given there were no foreign antigens to sensitize the recipient. However, for non-A1 recipients, the presence of A1 antigen on the donor heart may have resulted in the development of antibodies and subsequent adverse outcomes. We also expected subclinical evidence of an immune response in the form of DSA, which is a major risk factor for the development of AMR and CAV and a poor prognostic factor for mortality [11]. Although our study did not analyze this specific subgroup, the essentially equivalent outcomes of the match and mismatch groups make it very unlikely that they fared worse at one year post-HTx. Future studies should focus on A1 into non-A1 HTx and compare these with non-A1 into A1 and ABO-matched HTx.

We did find that the mismatch group had a higher hospital LOS after their HTx. This may have been driven by a higher incidence of PGD and vasoplegia. The mechanisms underlying PGD and vasoplegia are poorly defined; extensive research has revealed various donor, recipient, and intraoperative risk factors, although none of these are related to blood type mismatch [12,13]. It is unclear what would have caused an increase in these complications in our cohort given their similar pre-transplant characteristics. Further study is required to confirm our findings and determine their underlying mechanism.

Even though they had a higher hospital LOS, the mismatch group had similar one-year outcomes compared to the match group. Our study offers preliminary evidence that suggests A1-mismatched HTx can be performed safely. If future studies confirm our results, donor hearts can be more efficiently allocated. Currently, non-A1 recipients with anti-A1 antibodies often receive O organs [5]; this can prolong wait times for O recipients, which are already up to three times longer than for patients with other blood types [14]. Eliminating the requirement for A1 matching would decrease time on the transplant waitlist, especially for non-A1 and O recipients.

Another way to take advantage of the reduced immunogenicity of non-A1 subtypes is transplanting these organs across the ABO barrier [15]. This practice has already been established in kidney and liver transplantation. Group O and B recipients with low (<1:8) anti-A titers who received A2 kidneys have rates of transplant success similar to ABO-compatible transplants [16]. In 2014, the kidney allocation system expanded to allow transplantation of A2 and A2B kidneys into group B recipients resulting in a subsequent > 9% increase in kidney transplants among these patients [17]. Similar experiences have been reported in liver transplantation, with one registry analysis of O recipients showing no difference in retransplantation, rejection, graft survival, or overall survival when using A2 livers versus O livers [18]. As such, A2 to O liver transplantation has been promoted as a strategy to decrease long waitlist times for these patients [19]. Study on this topic in HTx is much more limited, with one case of an A2 heart transplanted into an O recipient reported in 1993. The patient was hemodynamically stable but retransplanted after only four days. Pathology did not reveal any cellular infiltrate or antibody deposition [20]. This strategy’s success in kidney and liver transplantation suggests that it would be viable in HTx as well; further study is needed to determine its benefits and risks.

An important caveat is that no study of A1-mismatched organ transplantation has found recipient anti-A1 antibody titers >1:8, including ours. Anti-A1 antibodies are rare, with a prevalence of 1% in A2 individuals. When they do exist, they are often non-reactive at body temperature and considered clinically insignificant [5]. Our study only measured anti-A1 antibody titer once. As the titer was measured at time of enrollment, there was a variable amount of time between HTx and titer monitoring between each subject. We were also unable to confirm or refute any possible association between anti-A1 antibody titers and HTx complications, such as PGD, vasoplegia, and rejection.

Given the increased LOS in the mismatch group, further study is needed to determine whether these associations exist. Such a study should include daily titer monitoring in the peri-HTx period as well as during episodes of suspected rejection. This would provide valuable information on the trajectory of anti-A1 antibody titers in response to HTx and its associated immunosuppression. Previous studies lacked consistency in checking titers before and/or after transplant and have not been able to provide this level of detail. Additional information regarding early post-transplant biomarkers, such as troponin and B-type natriuretic peptide, would also be useful. Rather than emphasizing A subtyping, more frequent titer monitoring may allow for its use in the prognostication of HTx outcomes, and potentially influence decisions on whether to perform HTx or modify immunosuppression.

A limitation of this study is that with an average of 2.3 years between HTx and enrollment, only 39% of our subjects were enrolled prior to the one-year mark. Subjects who had short-term post-HTx morbidity and mortality were therefore likely under-represented, resulting in reported outcomes that are better than expected. Although this effect would have had an equal impact on the match and mismatch groups, our study likely minimizes any possible effect that A1 mismatch may have had on adverse outcomes in the early post-HTx period. The increased LOS in the mismatch group already suggests higher short-term risk in these transplants. A fully prospective study with enrollment of all subjects prior to HTx is required to provide more data regarding early adverse events.

As we did not collect data beyond one year post-HTx, we were unable to fully assess the incidence of CAV, which increases with time and affects up to 50% of recipients at ten years post-HTx [21]. It is conceivable that non-A1 recipients may eventually develop antibodies against A antigen expressed by the allograft endothelium, resulting in the development of CAV. Studies with longer follow-up times and larger sample sizes are necessary prior to establishing A1-mismatched HTx as safe. 

## 5. Conclusions

Our study is the largest analysis of A1-mismatched HTx to date. Although it was limited by delayed enrollment of subjects and short follow-up time, our results provide an encouraging initial sign that A1-mismatched HTx does not result in worse outcomes at one year. Future study focused specifically on A1 into non-A1 HTx with a large cohort entirely enrolled prior to HTx and a longer follow-up interval is required to confirm the safety of A1-mismatched HTx. Emphasis should be placed on examining the increased hospital LOS that was found in our mismatch group. If future studies establish the safety of A1-mismatched HTx, elimination of the A1 barrier would reduce waitlist time, especially for non-A1 and O recipients.

## Figures and Tables

**Table 1:** Pre-Transplant Clinical Characteristics ^1^.

	A1 Donor-Recipient Match (*n* = 110)	A1 Donor-Recipient Mismatch (*n* = 32)	*p*-Value
Recipient Age (Years)	57.4 ± 12.9	55.2 ± 12.1	0.40
Donor Age (Years)	37.3 ± 12.8	34.9 ± 13.2	0.35
Race			0.02
White	80.9% (*n* = 89)	62.5% (*n* = 20)	-
African-American	12.7% (*n* = 14)	12.5% (*n* = 4)	-
Asian	3.6% (*n* = 4)	6.3% (*n* = 2)	-
Other	2.7% (*n* = 3)	18.8% (*n* = 6)	-
BMI (kg/m^2^)	25.1 ± 4.8	25.9 ± 4.2	0.41
PHM	1.08 ± 0.230	1.01 ± 0.163	0.10
Female	29.0% (*n* = 32)	25.0% (*n* = 8)	0.65
Type of Cardiomyopathy			0.63
Nonischemic	61.8% (*n* = 68)	65.6% (*n* = 21)	-
Ischemic	26.4% (*n* = 29)	21.9% (*n* = 7)	-
Congenital	0.9% (*n* = 1)	3.1% (*n* = 1)	-
Restrictive/Infiltrative	10.9% (*n* = 12)	9.4% (*n* = 3)	-
Previous Diabetes	33.6% (*n* = 37)	31.3% (*n* = 10)	0.80
Previous Hypertension	59.0% (*n* = 65)	53.1% (*n* = 17)	0.55
Prior Cardiac Surgery	41.8% (*n* = 46)	34.4% (*n* = 11)	0.45
Cytomegalovirus Mismatch	46.4% (*n* = 51)	59.4% (*n* = 19)	0.20
Previous Pregnancy in Females	65.6% (*n* = 21)	50.0% (*n* = 4)	0.44
Prior Blood Transfusion	30.9% (*n* = 34)	31.3% (*n* = 10)	0.97
Pre-transplant PRA with MFI > 5000	15.5% (*n* = 17)	6.3% (*n* = 2)	0.24
Pre-transplant Desensitization	9.1% (*n* = 10)	9.4% (*n* = 3)	1.00
Induction with ATG	58.2% (n = 64)	50.0% (*n* = 16)	0.41
Time on Waitlist (days)	149.7 ± 269.8	153.8 ± 254.4	0.94
Urgent Status at Transplant ^2^	66.4% (*n* = 73)	65.6% (*n* = 21)	0.94
Mechanical Circulatory Support	41.8% (*n* = 46)	43.8% (*n* = 14)	0.85
Pre-transplant Creatinine (mg/dL)	1.5 ± 0.9	1.6 ± 1.8	0.69
Ischemic Time (min)	181.7 ± 52.7	195.4 ± 44.0	0.18

^1^ Values listed as mean ± SD or as %. ^2^ Status 1A pre-2018 scheme change; Status 1, 2, 3 post-change.

**Table 2:** Post-Transplant Outcomes ^1^.

	A1 Donor-Recipient Match (*n* = 110)	A1 Donor-Recipient Mismatch (*n* = 32)	*p*-Value
Primary Graft Dysfunction	12.7% (*n* = 14)	18.8% (*n* = 6)	0.40
Vasoplegia	11.8% (*n* = 13)	15.6% (*n* = 5)	0.56
Intensive Care Unit Length of Stay (Days)	6.5 ± 3.6	8.0 ± 5.8	0.08
Hospital Length of Stay (Days)	13.5 ± 8.4	17.1 ± 10.2	0.04
Ejection Fraction (%) ^2^	62.6 ± 5.2	60.9 ± 4.7	0.11
Donor-Specific Antibodies ^2^	17.3% (*n* = 19)	9.4% (*n* = 3)	0.41
Survival ^2^	99.1%	100.0%	0.58
Freedom from CAV^2^	98.2%	96.9%	0.67
Freedom from NF-MACE ^2^	93.6%	90.6%	0.52
Freedom from ATR ^2^	76.4%	81.3%	0.61
Freedom from ACR ^2^	87.3%	90.6%	0.62
Freedom from AMR ^2^	91.8%	90.6%	0.80

^1^ Values listed as mean ± SD or as %. ^2^ Outcomes at one year post-transplant.

## Data Availability

The data presented in this study are available on request from the corresponding author. The data are not publicly available to protect patient privacy.
